# Synthesis, characterization and evaluation of new alternative ruthenium complex for dye sensitized solar cells

**DOI:** 10.1038/s41598-024-66808-1

**Published:** 2024-07-19

**Authors:** H. A. Fetouh, A. E. Dissouky, H. A. Salem, M. Fathy, B. Anis, A. E. Hady Kashyout

**Affiliations:** 1 Chemistry Department, Faculty of Science, Alexandria University, Alexandria, Egypt; 2 Electronic Materials Department, Advanced Technology and New Materials Research Institute, City of Scientific Research and Technological Applications (SRTA-City), Alexandria, Egypt; 3 Physics Division, Spectroscopy Department, National Research Centre (NRC), Dokki, Cairo Egypt

**Keywords:** Ruthenium complex, Dye sensitized solar cells, TiO_2_ Nano crystalline, Redox behavior, Time resolved, Chemistry, Materials science

## Abstract

For first time, new innovative ruthenium N3-Dye anchored with selenium (Se) and N3 dye anchored with sulphur atoms were synthesized in a good yield. Dyes are applied and evaluated in performance of dye sensitized solar cell. N3–Se dye showed superior photochemical& electrochemical behavior and high rate electron transfer across anode surface than N3–S dye. The better optical and electrochemical activities would make Se-dye a candidate for applications in solar cells. Half life time of N3–S showed a single exponential decay with an average lifetime of 0.8 ns. For N3–Se dye, decay curve was fitted by sum two exponential functions with 75% and 25% counts have 2.5 ns and 30 ns respectively. Solar cells were fabricated and analyzed to determine their solar-to-electric conversion efficiency under standard AM 1.5 sunlight. Commercial N3 dyes showed current density (J_sc_) of 17.813 mA cm^−2^, open circuit potential (V_oc_) of 0.678 V, filling factor (FF) of 0.607 and conversion efficiencies (η) of 7.3%. Corresponding values for N3–S dye, Jsc 11.2 mA cm^−2^, V_oc_ 0.650 V, FF 0.681 and η 5%. Se–N3 dye, showed J_sc_ = 6.670 mA cm^−2^, V_oc_ = 0.6004 V, FF = 0.77 and η = 3.09%. Long lifetime of N3–Se caused low practical performance.

## Introduction

International energy agency recently^1^ announced that solar photovoltics (PVs) as the cheapest source of energy production than new coal-or gas-fired power plants in most countries and solar energy is the lowest cost electricity ever seen. Solar energy is a promising energy source as it is clean and zero cost energy resource with consideration of technology costs for building up energy convertors either solar photovoltics (PVs), concentrated solar power, or solar thermal. PVs convert solar energy into electricity by photovoltic effect. Commercially, standard mono-, and polycrystalline silicon solar panels have power conversion efficiencies around 24%, however, these semi-conductor silicon solar cells have high prices^2^.

Dye-sensitized solar cells (DSSCs) are promising photovoltic solar cells simply fabricated at low production cost and wide indoor applications^3^. In DSSCs, organometallic or organic dyes molecules adsorbed on surface of mesoporous Nano crystalline metal oxide films to convert sunlight into electricity. Ruthenium (Ru) complexes are common photosensitizers stable in oxidized state. Moreover, Ru is photo-electrochemically active^4–7^.

DSSCs used Ru(II)-polypyridyl complexes as organometallic dye has overall certified power conversion efficiency (PCE) 12.3%. Lab-scale PCE has reached 14.3% under standard (Global Air Mass 1.5) illumination^8^. Wide absorption range from visiable to nearinfrared (NIR) and carboxylate COOH groups attached to bipyridyl moiety lower the energy level of π* orbitals of the ligand. Absorption spectra of Ru-polypyridyl complexes can be tailored by adjusting energy levels of the highest occupied molecular orbital (HOMO) and the lowest unoccupied molecular orbital (LUMO) of these photosensitizing dyes.

The ideal sensitizer PVs cell converting standard global AM1.5 sunlight to electricity should: absorb all incident light below threshold wavelength 920 nm^9^, has electron donors functional groups like either carboxylate or phosphonate grafted on surface of semiconductor oxide such as TiO_2_. After electronic excitation from ground state, electrons injected into surface of TiO_2_ with unity quantum yield^3^. Energy level of the excited state should be matched to the lower bound of conduction band of TiO_2_ to minimize energy loss during electron transfer. redox potential of the dye should be sufficiently positive for regeneration by electrons from redox conductor electrolyte. It should be stable enough to sustain about 10^8^ turnover cycles corresponding to about long period of exposure to sun light^9^.

Different types of dye structures and Ru complexes have been applied as photosensitizers in DSSCs, due to the unique photo electrochemical properties, stability and many oxidation states range from I to IV, and formation of very inert chemical bonds with imine-nitrogen centers^10–14^. Se-Ruthenium (Ru) photosensitizer in DSCC showed no reported study. The current study aims to: preparation, characterization and evaluation of efficient Ru dye [Ru(II)(4,4′-(CO_2_H)2-2,2′-bipyridine)_2_(NCS)_2_] abbreviated as N3–S for DSSCs and a modified dye by introducing Se atom instead of sulphur atom giving novel alternative dye N3–Se dye. Investigation of the physicochemical properties of dyes, electrochemical behavior; efficiency in DSSCs and comparison behavior of the dyes under irradiation by standard global AM1.5 sunlight will be illustrated in details.

## Materials and methods

All chemicals used in this study have been purchased from SIGMA ALDRICH and used without further purification: Anhydrous ruthenium (III) chloride; N,N-Dimethyl formamide (anhydrous 99.8%); 2,2′-bipyridyl-4,4′-dicarboxylic acid (98%); ammonium thiocyanate (97.5%); potassium selenocyanate (99%); anhydrous sodium hydroxide; nitric acid (HNO_3_); acetonitrile (99%), absolute ethanol (99%), lithium iodide (99.999%); iodine (99%), 3-methoxyproponitrile (> 99%); 4-tertbutylpyridine (99%); titanium isopropoxide (99.999%); Acetic acid (99.8%); isopropanol (99.8%); Triton X-100 (99%); acetone (99%) and tetra-butyl-ammonium-hexafluoro-phosphate (98%). FTO-glass sheet of electrical resistance 7 Ω/□ is used for fabrication of the PV cells. Anode electrode is high transmittance conducting glass coated by layers of TiO_2_ nanoparticles and counter cathode electrode is Pt.

Cis-[RuL_2_(NCS)_2_): Cis-Bis(4, 4′-dicarboxy-2, 2′-bipyridine)di-thio-cyanato Ru(II) complex abbreviated ascis-[RuL_2_(NCS)_2_] (L is (2, 2′-Bipyridyl-4,4′-dicarboxylic acid) was synthesized following reported modified method (K. Wongkhan et al*.*) with definite precursor concentrations, reaction temperature and reaction time^15^. A weight of (250 mg, 0.95 mmol) RuCl_3_·3H_2_O and 2,2′-bipyridine-4,4′-dicarboxylic acid (475 mg, 1.925 mmol) were dissolved in 50 mL anhydrous dimethyl formamide (DMF).

**Figure 1:**
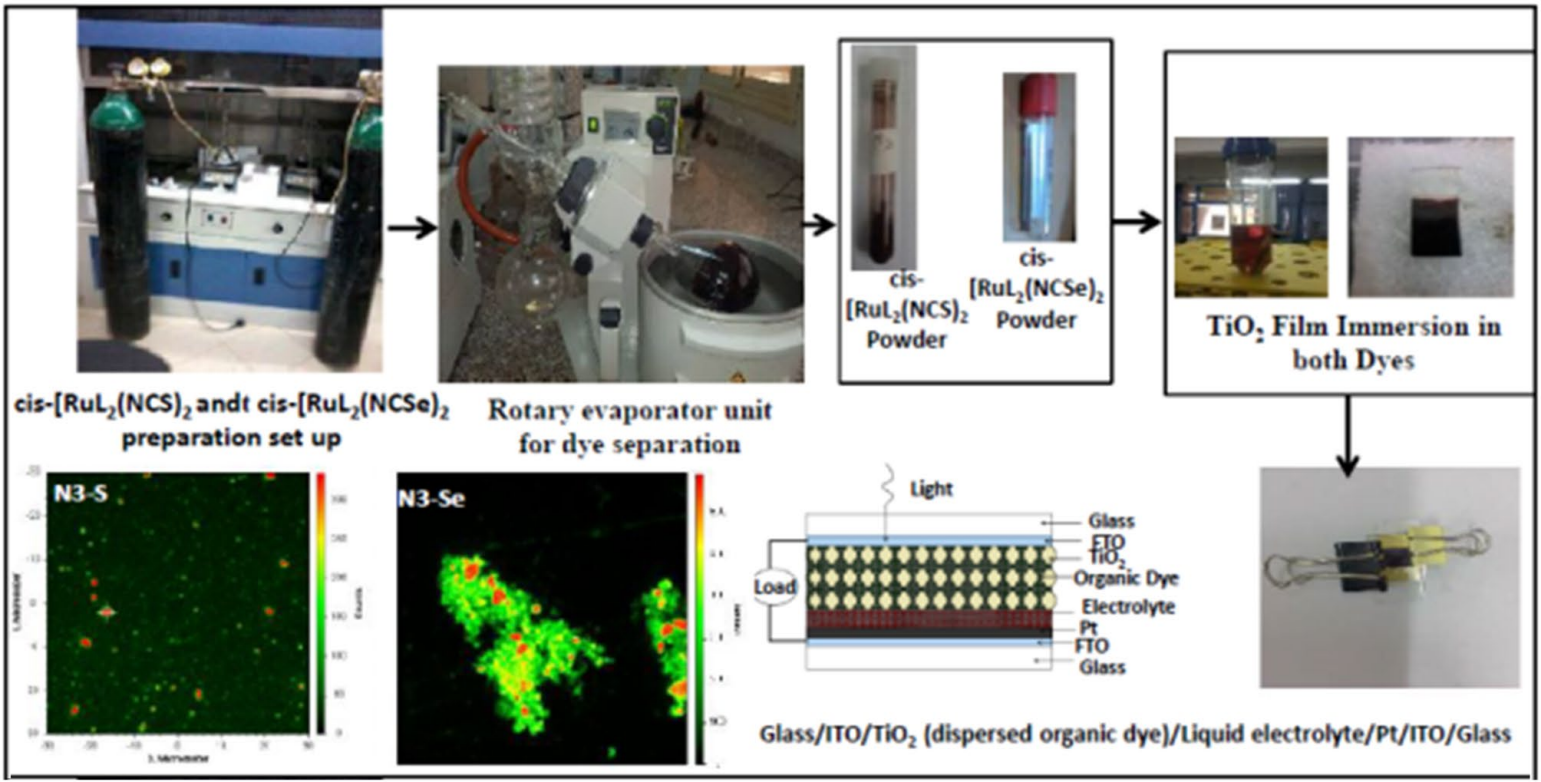
Flow diagram illustrating synthesis of Ru dyes and fabrication of DSSCs.

Reaction mixture was heated under reflux in a nitrogen atmosphere at 140 °C for 7 h, then slowly cooled to the room temperature. Solutions of 0.1 M NaOH (8.3 mL, 0.82 mmol) and ammonium thiocyanate (0.325 g, 4.325 mmol) were added to the reaction mixture that heated under reflux for 4 h. DMF solvent was removed using a rotary evaporator at 60 oC under vacuum giving a red-dark residue that is dissolved in double distilled water acidified by 0.1 M HNO_3_ to adjust solution acidity to pH 2. Precipitate was filtered again. Red-dark crystals (1.2 g, 83% yield) obtained. Process preparation and application of dyes in DSSCs are shown in Fig. 1.

[RuL_2_ (NCSe)_2_] abbreviated as N3–Se had been synthesized following synthesis method of N3–S dye. NH_4_SeCN is added instead of NH_4_SCN in an appropriate amount. Dye color is dark brown crystals (1.2 gm., 83% yield).

DSSC design included working electrode (TiO_2_ film with 8–10 µm thickness). TiO_2_ is sensitized with either N3–S or N3–Se dyes. A counter platinum electrode and electrolyte solution are prepared. Working electrode is fluorinated tin oxide conducting glass sheet (FTO) with an electrical resistance 7–15 Ω/□ cleaned in an ultrasonic bath for 15 min. in double distilled water and acetone, respectively. TiO_2_ paste was prepared by mixing anatase TiO_2_ nano powder^16^ with a few drops of acetylacetone and Triton X-100 surfactant. Using doctor blade technique; successive four layers of TiO_2_ paste were spread on the surface of FTO glass substrate, air dried for 20 min., then heat treated at 450 °C for 30 min., and cooled to 80 °C. For sensitization; TiO_2_ anode electrodes were immersed in 5 × 10^−5^ M ethanolic solution of N3–S or N3–Se dye for 24 h. Anode surface was rinsed with ethanol to remove excess dye particles from its surface.

Counter cathode electrode is thin film platinum deposited on FTO glass substrate using sputtering technique (Hummer 8.1-USA) (R.F power 200 W for 5 min.)^17^. Deposits annealed at 400 °C for 15 min. Two drops electrolyte solution: 0.5 M LiI, 0.05 M iodine I_2_, 3-methoxypropionitrile, and 0.5 M 4-tertbutyl pyridine have been inserted between working-, and counter electrodes, which are tightly closed using clamps as showed in Fig. 1.

**Figure 2:**
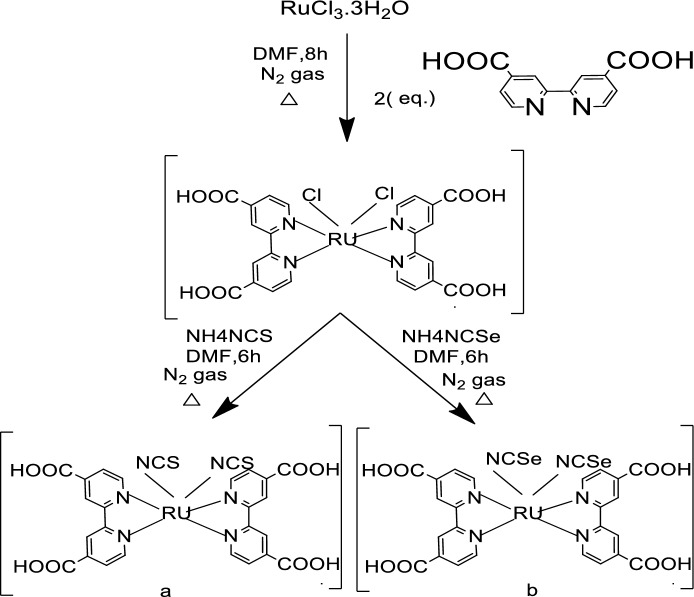
Reaction synthesis routes: (**a**) [RuL_2_) (NCS)_2_] (N3–S), (**b**) [RuL_2_) (NCSe)_2_] (N3–Se).

Ru(II) complexes are synthesized according to Fig. 2. These semi organic materials are air stable at the room temperature for a long time, soluble in absolute ethanol and DMF but sparingly or insoluble in acetonitrile or water. Chemical structures of the dyes were established utilizing C, H, N elemental analysis, FTIR, UV/Vis., photoluminescence (PL), and mass spectral analysis. The electrochemical behavior and half life times were also discussed.

FTIR spectra of the prepared dyes were recorded using KBr discs at frequency range 100–4000 cm^−1^ using Fourier Transform Infrared (FTIR) spectrophotometer (Shimadzu FTIR-8400 S, Japan). CHNS analysis was determined using Perkin-Elmer 240B elemental analyzer, USA. Atomic percentage of Ru(II) ion was determined using atomic absorption spectroscopy (AAS) technique. Nonlinear optical activity of 10^–5^ M ethanolic solution dyes was investigated using UV–Vis.-1800 PC, Shimadzu, Japan) spectrophotometer.

Photoluminescence (PL) activities of the dye were studied using fluorescence spectrometer (Perkin-Elmer model IS 55, USA). Mass spectra were recorded by electron ionization technique at 70 eV using Thermo scientific LTQ mass spectrometer (Thermo Fisher Scientific, USA) to determine molecular weight of the dyes. Fluorescence lifetime images were acquired using FLIM system (ALBAv5 from ISS, Japan) on excitation using laser diode 650 nm coupled with scanning module (ISS) through multiband dichroic filter to Epifluorescence microscope (Olympus, Model IX73) with UPLSA 60X objective 1.2 NA, 0.28 mm wd. Emission was observed and detected by cooled low noise (below 100 counts/sec.) timecorrelated single photon counting (TCSPC) detector GaAs fast PMTs. FLIM data were acquired using ISS A330 fast FLIM module with n harmonics 20 M Hz laser repetition frequency. FLIM data were analyzed with Vista Vision Suite software (Vista v.204 from ISS). FLIM analysis was carried out using fitting algorithm and phasor analysis. Electrochemical behaviors of the dyes were studied using electrochemical impedance spectroscopy (EIS) at open circuit potential and cyclic voltammetry using Gamry Potemtiostat (USA) reference 600 working by sequencer software version 6.20 setup. Three-neck electrochemical cell contained: silver/silver chloride reference electrode (RE), Pt counter electrode (CE), and graphite working electrode (WE) were connected to the potentiostat. Surface of WE was activated by paste of Al_2_O_3_-double distilled water. Software Gamry Echemanalyst version 6.20 was used for analysis of Nyquist impedance plots and cyclic voltammograms^18^. Current–voltage (I–V) curves were recorded using solar simulator vice (PET Photo Emission Tech., Inc. USA) under illumination using standard global AM1.5 sunlight measurements.

### Ethics approval and consent to participate

No ethical issue . All authors approved participation consent.

## Results and discussion

Elemental analysis collected in Table 1 confirmed the postulated chemical formula for dyes [RuL_2_X_2_], where X = NCS^−^ or NCSe^−^.

**Table 1:** Elemental analyses data and some physical properties of Ru dyes.

Dye	Color	Element analysis %Cal. (% measured)				
		%C	%H	%N	% S(Se)	%Ru
[RuL_2_(NCS)_2_	Dark red	44.25	2.29	11.90	9.09	14.33
		(44.26)	(2.28)	(11.90)	(9.09)	(14.34)
[RuL_2_(NCSe)_2_	Dark brown	39.06	2.02	10.51	19.75	12.65
		(39.05)	(2.03)	(10.52)	(19.74)	(12.66)

The calculated % and measured values of the elemental analysis are in good agreement with a slight differences as shown in Table 1.

**Figure 3:**
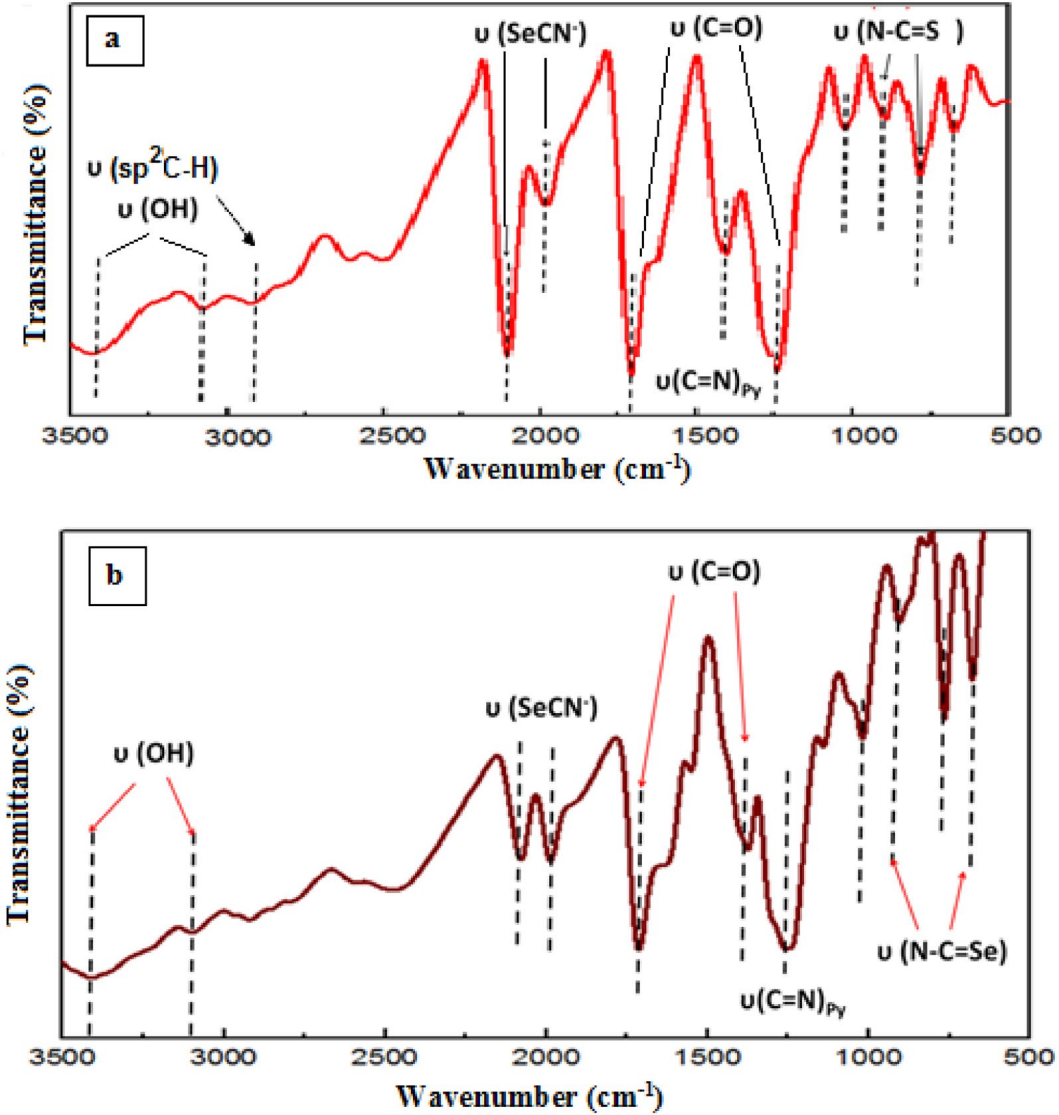
(**a**) FT-IR spectra of [cis-[RuL_2_ (NCS)_2_] and (**b**) [cis-[RuL_2_ (NCSe)_2_] respectively.

FTIR spectra of dyes [RuL_2_X_2_] are shown in Fig. 3a,b. The main IR vibrational bands with their tentative assignments are given in Table 2. Two bands at 3433 cm^−1^ and 3078 cm^−1^ (S–N3) dye at 3407 cm^−1^, 3066 cm^−1^ (Se–N3) for stretching υ(OH). Band at 2923 cm^−1^, 2908 cm^−1^ for υ(sp^2^ C–H) for SCN^−^, SeCN^−^ respectively. Absorption bands at 1708 cm^−1^ 1713 cm^−1^ for SCN^−^, SeCN^−^, respectively assigned υ_C=O_ of protonated carboxylic COOH^+^_2_ group. Bands at 1369 cm^−1^, 1363 cm^−1^ in SCN^−^ and SeCN^−^ respectively (υ_C=N_) of pyridine moiety. Doublet with peaks at 2105 cm^−1^, 1980 cm^−1^ for SCN^−^; 2079 cm^−1^, 1988 cm^−1^ for SeCN^−^ characterizing cis-configuration of two-thiocyanate or selenocyanate. NCS^−^ or NCSe^−^ group has two characteristic vibrational modes, υ_N=C_, υ_C=X_, (X = S or Se) diagnose coordination mode of ambidentate SCN^−^ or SeCN^−^ ligands^19,20^. N-coordination of thiocyanate or selenocyanate group is confirmed by the presence of υ_C=S_, υ_C=Se_ vibrational at 780 cm^−1^, 766 cm^−1^ respectively. Thiocyanate or selenocyanate groups coordinated to Ru through S or Se atoms. Weak stretching vibration band υ_C=S_ & υ_C=Se_ appeared at 700 cm^−1^^21,22^.

**Table 2:** Main IR bands (υ', cm^−1^) with their tentative assignments.

Complex	υ_OH_	υ _(sp2C-H)_ X = SCN^−^ SeCN^−^	υ (XCN),X = S/Se	υ(C = N)_Py_	υ (N–C = X) X = S/Se	υ_(C=O)_
[RuL_2_(NCS)_2_]	3424, 3079	2923	2105, 1980	1369	780, 700	1708, 1250
[RuL_2_(NCSe)_2_]	3407, 3066	2902	2079, 1988	1362	766	1713, 1252

**Figure 4:**
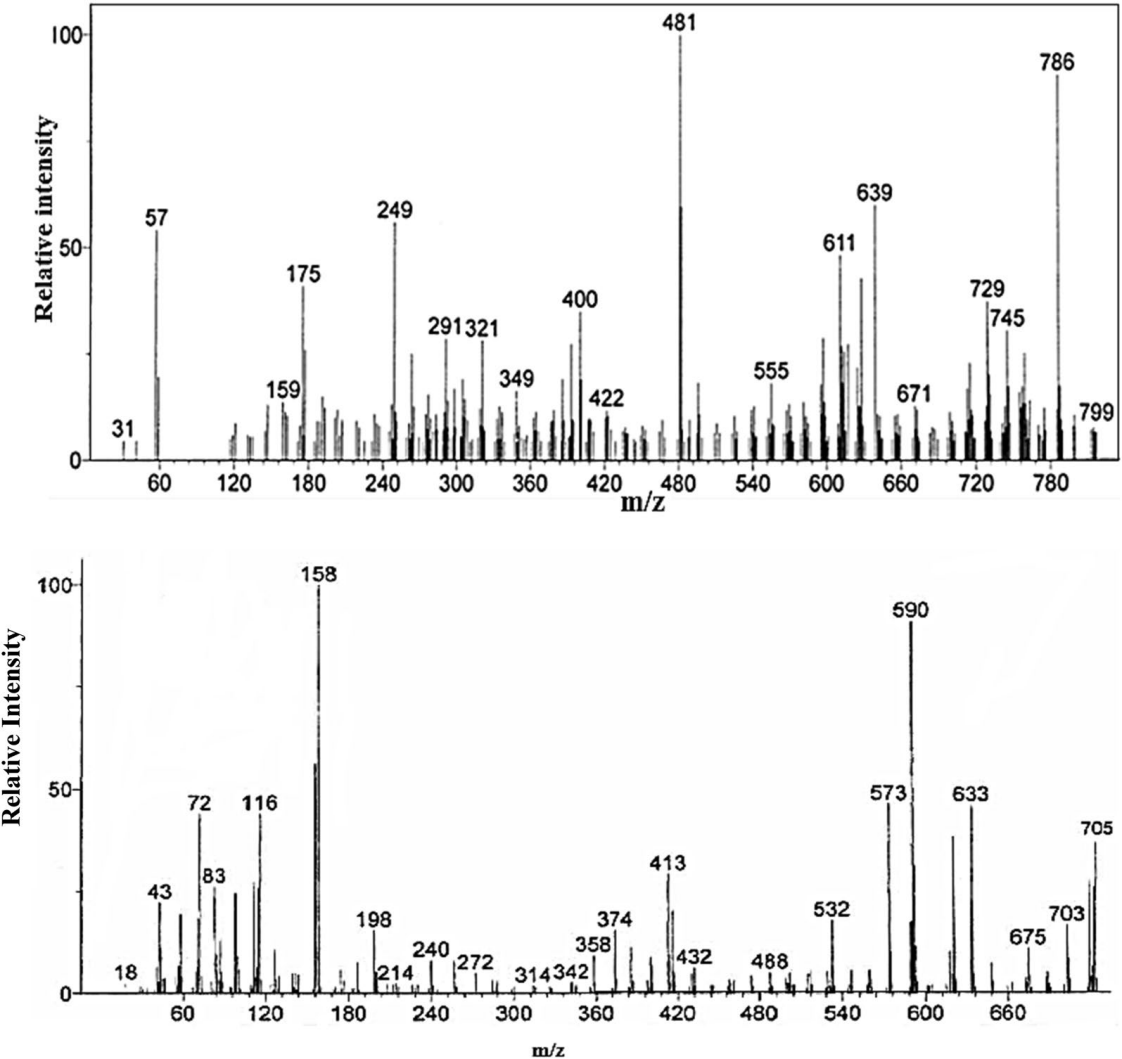
Mass spectra: cis-[RuL_2_(NCS)_2_] and cis-[RuL_2_(NCSe)_2_] respectively.

Molecular structures and chemical compositions of the dyes are confirmed from mass spectra, Fig. 4. Molecular ion signal as function of *(m/z)* giving molecular weights of dyes at the last spectral line.

**Figure 5:**
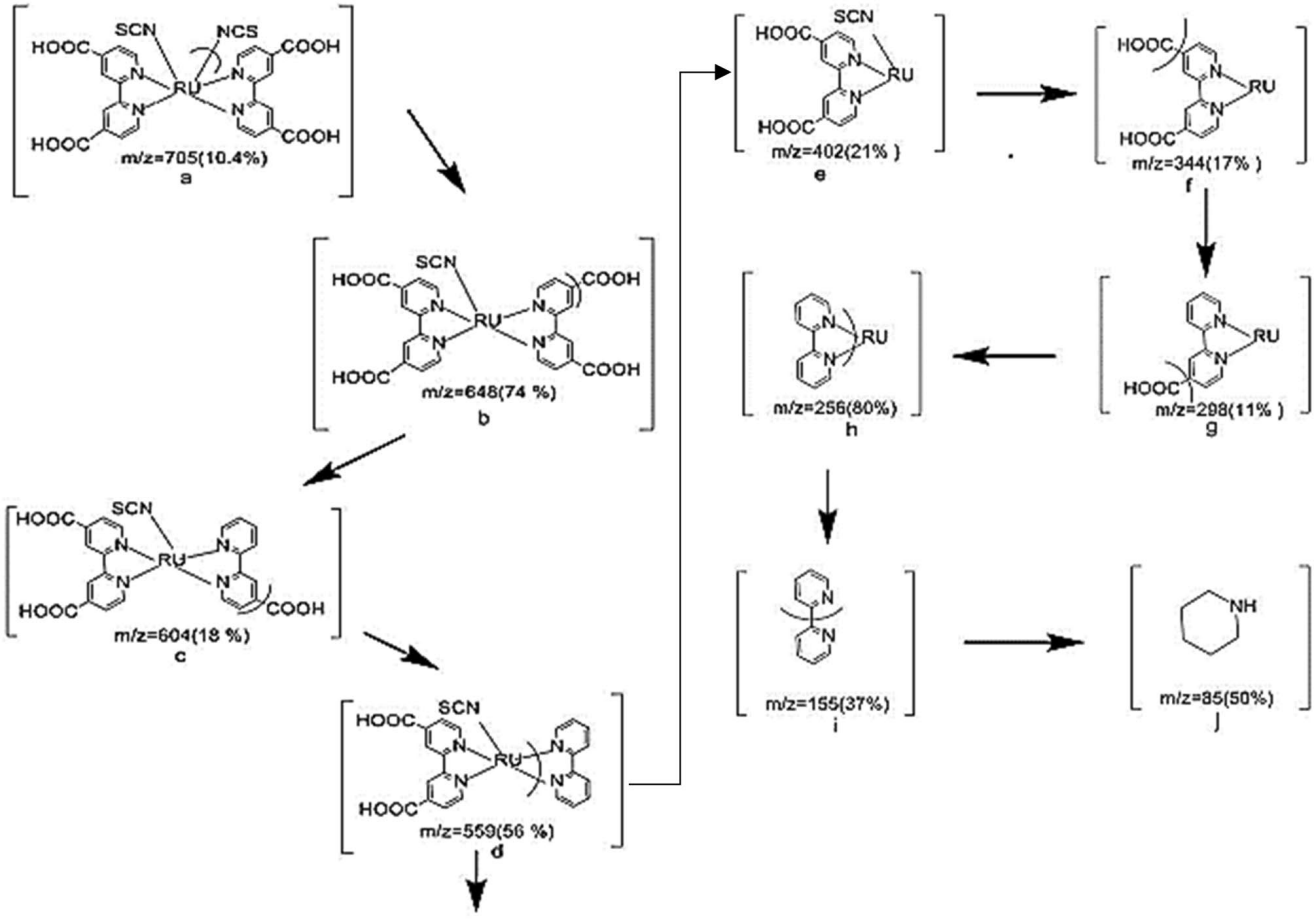
Proposed conductive fragmentation pathway for [RuL_2_(NCS)_2_].

Dye molecules are ionized into charged molecular ions on bombardment by electron beam that break some sample's molecules into charged fragments ions separated according to *(m/z)* ratio of mass spectra of [RuL_2_ (NCX)_2_], X = S or Se. Based on fragments ions, Fig. 5 is postulated for fragmentation of the dye molecules. Peaks at 705, 799 for X = S, Se, Main fragments *(m/z)* with their tentative assignments are given in Table 3.

**Table 3:** Relative intensities of the major molecular ions in Ru complexes.

[RuL_2_(NCS)_2_]			[RuL_2_(NCSe)_2_]		
Mass (m/z)	(%)	Fragment	Mass (m/z)	(%)	Fragment
705	10.4	a	799	10.4	a
648	74	b	697	54	b
604	18	c	653	50	c
559	56	d	609	86	d
402	21	e	453	72	e
344	17	f	349	16.3	f
298	11	g	304	54	g
256	80	h	259	54	h
155	37	i	159	13.6	i
85	50	j	85	50	j

Fragmentation pattern indicated dyes formed as [RuL_2_ (NCX)_2_]^23^. Mass spectra of the dyes are in good agreement with elemental analysis and FTIR spectral data and confirmed the formulation of dyes.

The new Se–N3 dye followed the same proposed fragmentation pathway except that S atom replaced by Se atom.

**Figure 6:**
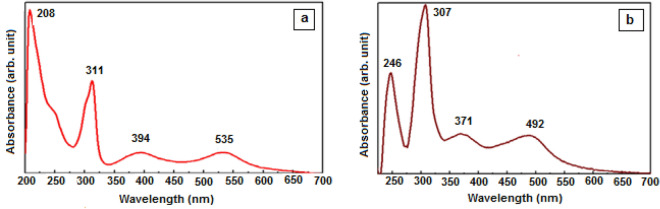
UV–Vis. spectra: (**a**) [cis-[RuL_2_(NCS)_2_], (**b**) [cis-[RuL_2_(NCSe)_2_].

UV–Vis. absorption electronic spectra of the dyes were measured in the ethanolic solution of the two complexes RuL_2_(NCS)_2_] and RuL_2_(NCSe)_2_] and were shown in Fig. 6. Intenses absorption band ligand-centered (LCT) π–π* transitions at 311 nm, 307 nm respectively with transition energy analogous to those of similar of Ru(II) polypyridine and agreed with transition energy reported for N3 dye^24–26^. Both complexes displayed MLCT bands at 350 to 600 nm range^27^. [RuL_2_(NCS)_2_] exhibits two broad bands at 394 nm and 533 nm. The bands for [RuL_2_(NCSe)_2_] at 371 nm, and 492 nm are attributed to MLCT absorptions. MC appeared as a shoulder for LC which disappeared in spectra of both complexes because of forbidden transition because it is d–d transition and may be obscured under LC band. Spectra of both complexes exhibit an intense band at 208 nm and 246 nm due to LMCT. Table 4 shows two absorptions in both dyes are red-shifted compared with that of [Ru(bpy)_3_]_2_^+^^28^ due to: distortion by existence of two 2,2′-bipyridine-4,4′-dicarboxylic acid molecules, two X = NCS^−1^ or NCSe^−1^ instead of three bipyridine molecules in each complex, and replacement of one strong bipyridine ligand by two weaker ligand molecules NCS^−1^ or NCSe^−1^ decreased charge transfer energy. Red shift of MLCT in N3–S relative N3–Se is due to stronger distortion in Ru-NCS^−^ complex than Ru-NCSe^−^. Electronic transition in [RuL_2_(NCSe)_2_] dye is more probable than that of [RuL_2_(NCS)_2_] dye due to the wide absorption bands at 371 nm, 492 nm.

**Table 4:** Electronic absorption spectral data for Ru complexes.

[RuL_2_(NCS)_2_]			[RuL_2_(NCSe)_2_]		
Λmax	ε (mol^−1^.L.cm^−1^)	Assignment	λ_max_	ε (mol^−1^.L.cm^−1^)	Assignment
208	162,000	LMCT	246	17,650	LMCT
311	91,900	LCT	307	29,550	LCT
394	20,200	MLCT	371	7050	MLCT
535	21,500		392	6750	MLCT

**Figure 7:**
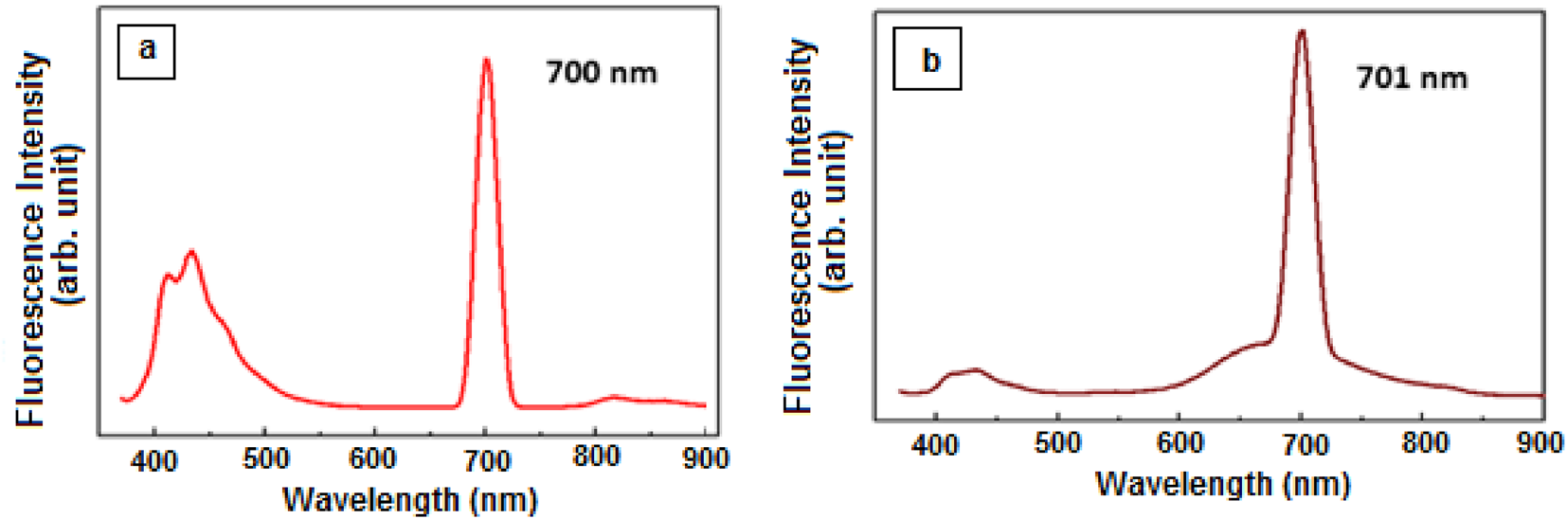
Photoluminescence spectra: (**a**) N3-dye, (**b**) N3–Se dye.

Figure 7a,b shows the photoluminescence properties of the Ru(II) polypyridine complexes dissolved in absolute ethanol, where (a) is the N3-dye and (b) is the N3–Se dye. The measurements were carried out with Spectro fluorometric characteristics: Emission spectra at an excitation wavelength of 380 nm.

Emission occurs from lowest-lying triplet ^3^MLCT excited state^29–31^. Emission bands at λ_max._ 700, 701 nm for [RuL_2_(NCS)_2_] and [RuL_2_(NCSe)_2_] respectively. Broad absorption band in UV–Vis. electronic absorption spectra showed that complexes have inter molecular charge transfer characteristics. Conjugation makes tuning between LUMO and HOMO energy levels in excited state and increases resonating structures of pyridine rings^25^.

Comparing between the dyes, the wider PL band for N3–Se dye at 700 nm indicated that it has more efficient PL activity than N3–S dye.

**Figure 8:**
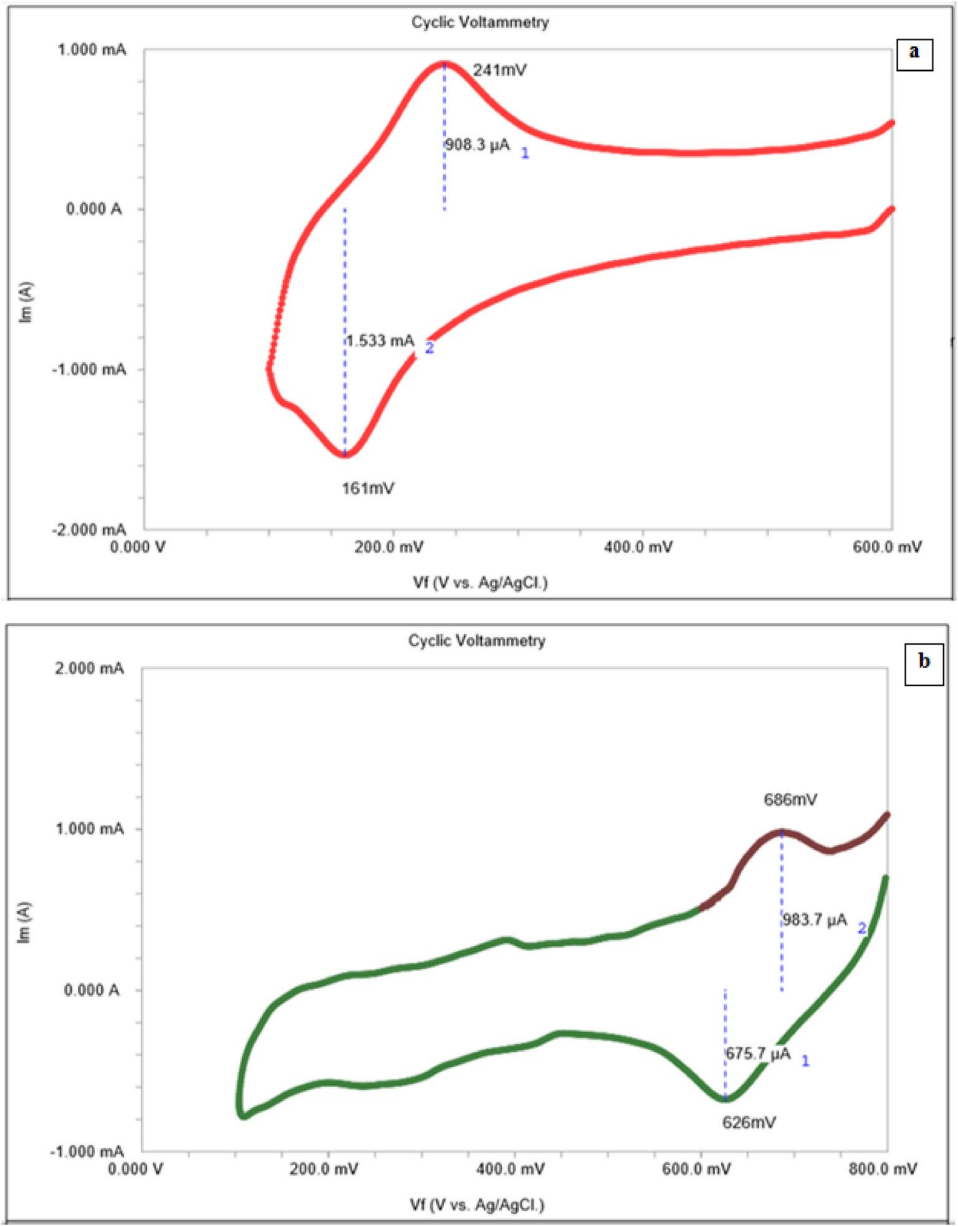

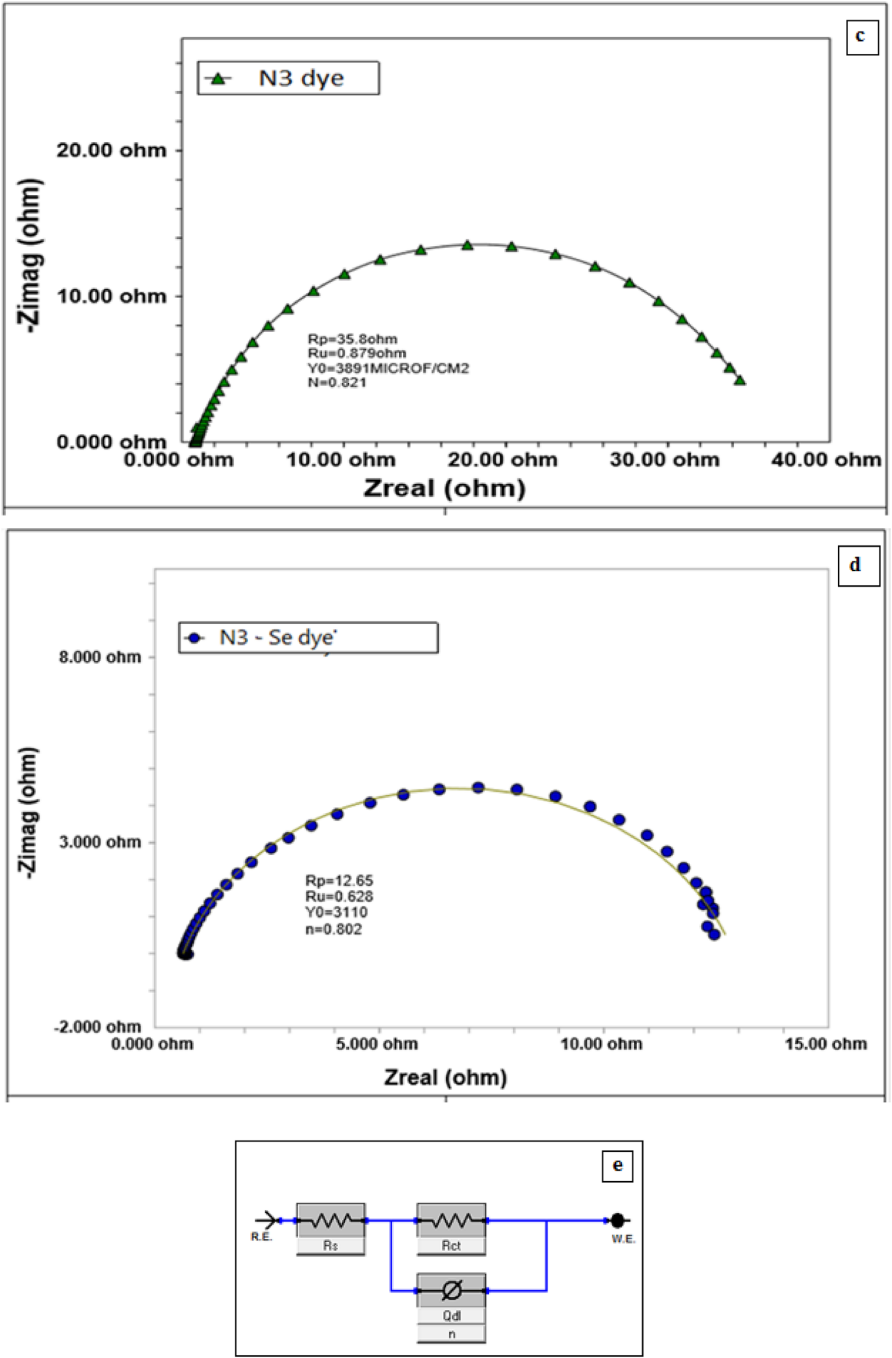
Cyclic voltamograms: (**a**) N3–S dye, (**b**) N3–Se Dye; (**c**,**d**) Nyquist plots of N3 dye, N3–Se dye respectively, (**e**) Equivalent circuit model.

Redox and electrochemical behavior of the dyes were clarified via cyclic voltammetry (Fig. 8a,b). Dyes are reversibly reduced on cathodic polarization and are oxidized on anodic polarization. Reversible redox behavior of dyes confirmed optical activity by donating lone pair electrons to TiO_2_. Single redox wave in cyclic voltammogram indicated high purity of the dyes^32^. Reversible cyclic voltamograms indicated that both dyes can be regenerated after oxidation. Table 5 shows polarization parameters for both N3 and N3–Se dyes. Dyes exhibited a signal reversible electrochemical wave over examined potential range with oxidation potential, E_1/2_ 0.241 V for N3-dye and E_1/2_ 0.686 V for Se-dye.

**Table 5:** Polarization parameters for N3–S and N3–Se dyes.

Dye	Molarity (M)	E_a_ (V)	E_c_ (mA)	∆E_P_	I_a_	I_C_	∆I_P_
N3–S	5*10^−4^	0.241	0.161	0.08	0.908	1.533	0.857
N3–Se		0.986	0.626	0.06	0.984	0.676	0.308

Under experimental conditions used in recording cyclic voltammograms, value of parameter $\Delta {E}_{P}$ represented ΔE_LUMO–HOMO_ in eV^33^. Se-dye has lower ΔE_LUMO–HOMO_ than N3dye. The much lower ∆IP for Se-dye indicates that nearly reversible equilibrium behaviors of this dye rather than N3 dye i.e. Se-dye is easily regenerated more than N3-dye. Adsorption strength of N3-dye and Se-dye on TiO_2_ electrode can be represented in Nyquist impedance diagrams (Fig. 8c,d). Impedance parameters (Table 6) were obtained via nonlinear fitting of Nyquist plots to the theoretical equivalent semicircle model shown in Fig. 8e ^34^.

**Table 6:** Impedance parameters for N3–S and N3–Se dyes.

Dye	Molarity	R_ct_ ohm.cm^2^	R_s_	n	Q_dl_ µF cm^−2^
N3–S	5*10^−4^	35.8	0.88	0.821	3891
N3___Se		12.69	0.63	0.80	3110

Resistance of solution and adsorbed film of dye molecules is represented by element R_s_, Resistor, R_ct is_ connected parallel to capacitor, Q_dl_ represents capacitance of electric double layer (edl) at electrode/solution interface. System heterogeneity is represented by parameter n. Electron transfer resistance from dye to TiO_2_ across surface of the working electrode is represented by charge transfer resistance (R_ct_). The rate of electron transfer equals reciprocal 1/R_ct_.

**Figure 9:**
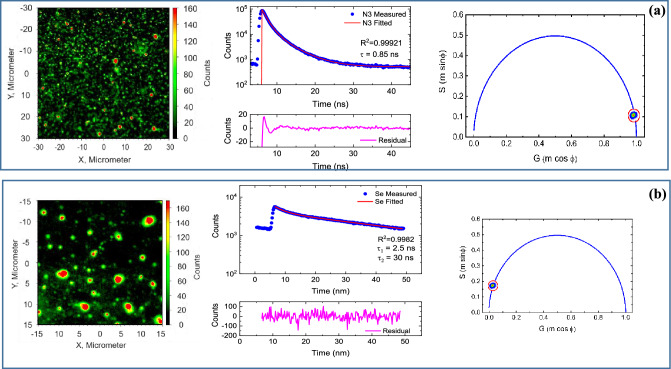
Raw FLIM data: (**a**) N3–S, (**b**) N3–Se dye. Middle panel shows PL intensity decay curve of each dye with fitting curve. Curve at the bottom panels is the fitting residual. Left panel in a, b is phasor plot representations from fluorescence FLIM data.

For lifetime measurements for N3–S and N3–Se Dyes, Fig. 9a shows raw FLIM data for N3–S dye, with PL intensity decay curve fitted by sum of two exponential functions with more than 98% of counts have 0.85 ns half lifetime. The right panel of Fig. 9a shows phasor plot Intensity decays for corresponding FLIM image was shown in left panel of Fig. 9a. In phasor plot, each pixel in FLIM image is represented in 2D diagram with two coordinates S and G (given by Eqs. 1, 2) based on phase shift (φ) between transmitted wave and resulting PL wave and demodulation factor (m) in laser source^35^:

1 $${\text{S}} = {\text{m}}\;{\text{sin}}\left( \varphi \right) = \omega \tau /\left( {{1} + \omega^{{2}} \tau^{{2}} } \right)$$

and

2 $${\text{G}} = {\text{m}}\;{\text{cos}}\left( \varphi \right) = {1}/\left( {{1} + \omega^{{2}} \tau^{{2}} } \right)$$

where τ is lifetime, ω = 2πf is laser modulation angular frequency (20 MHz). From Eqs. (1, 2),

3 $$\tau = \omega \left( {{\text{S}}/{\text{G}}} \right)$$

Phasor plot for N3–S dye showed point clusters located at edge of phasor plot semicircle, indicating that N3–S dye decay is single exponential decay with an average τ 0.8 ns as calculated from last equations.

For N3–Se dye (Fig. 9b), decay curve was fitted by sum of two exponential functions with 75% counts have 2.5 ns, 25% counts have 30 ns lifetime. Phasor plot shows relatively broad point clusters at left side of the circle, indicating that the decay is multi-components exponential decay.

From light current–Voltage. Characteristics for the Fabricated DSSC clarified as follows:

Efficiency (η) of DSCC is calculated using Eq. (3);

4 $$\eta = {\text{J}}_{{{\text{sc}}}} {\text{V}}_{{{\text{oc}}}} {\text{FF}}/{\text{I}}_{0}$$

where I_0_ is the incident irradiation power, J_sc_ is the short circuit current density (current density corresponding to V = 0), V_oc_ is open circuit voltage (voltage corresponding to zero current density, J_sc_ = 0), and FF is fill factor.

**Figure 10:**
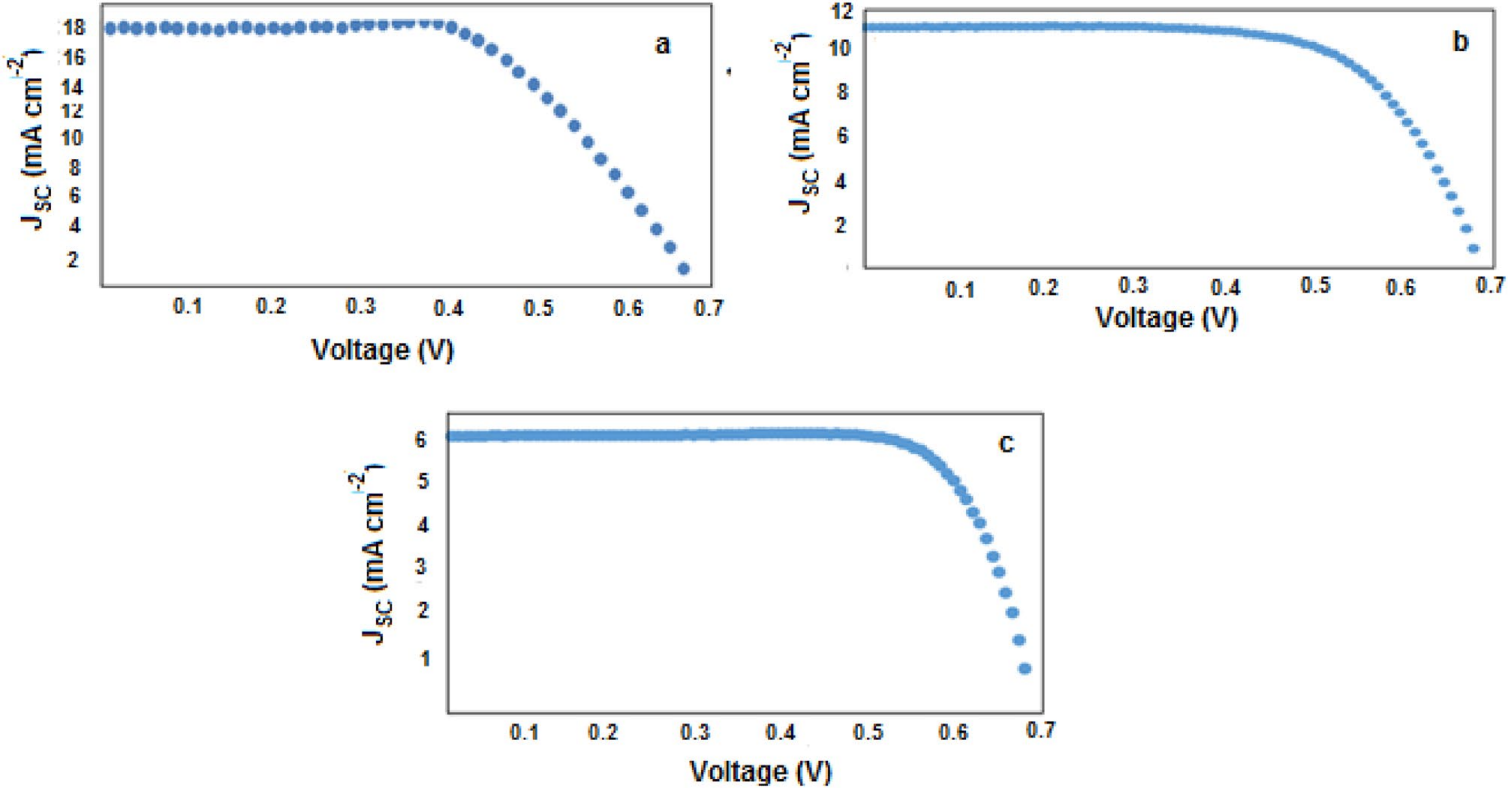
Current–voltage characteristics of solar cell using: (**a**) commercial N3 dye, (**b**) Prepared N3 dye, and (**c**) N3–Se dye.

Figure 10a–c showed cell I–V curves for DSSCs and the cell parameters when using commercial N3, prepared N3–S and N3–Se dyes, respectively.

Table 7 collected the cell parameters: For commercial N3 dye purchased from solaronix, the measured photovoltaic parameters are: J_sc_ = 17.813 mA/cm^2^, V_oc_ = 0.678 V, FF = 0.607 and η = 7.3%. For the prepared N3–S dye, the measured photovoltaic parameters areJ_sc_ 11.2 mA/cm^2^, V_oc_ = 0.650 V, FF = 0.681 and η = 5%. For N3–Se dye, the measured photovoltaic parameters are J_sc_ = 6.67 mA/cm^2^, V_oc_ = 0.6004 V, FF = 0.77 and η = 7.30%. Results indicated a comparable high cell performance for the commercial and prepared N3 dyes. For N3–Se dye which is prepared for the first time, it indicates a good cell performance with an efficiency of about 3.09%.

**Table 7:** Output cell parameters for sensitizers dyes: commercial N3, prepared N3–S and N3–Se respectively.

Dye Name	Cell area	Voc (V)	Jsc (mA/cm2)	F.F	η (%)
Commercial	0.25 cm^2^	0.675	17.813	60.73	7.3
N3 Prepared N3–S		0.650	11.2	68.06	5
N3–Se dye		0.6004	6.670	77.29	3.09

## Conclusion

Two sensitizers based on ruthenium complexes have chemical formula [RuL_2_(NCX)_2_], where L = 2, 2′-bipyridine-4,4′-dicarboxylic acid, X = S or Se are successfully synthesized. There is an excellent agreement between experimental and predicted spectra. N3–S exhibited enhancement of light harvesting in the red region and open up the way to improve significantly the overall efficiency of nanocrystalline photovoltaic devices than N3–Se. Cyclic voltammograms reflected high purity prepared Ru complexes. Excited-state oxidation potentials were determined and calculated via studying electrochemical behavior of these complexes. Determination of excited-state oxidation potential and lifetime of two sensitizers control electron-transfer process indicate the good performance of the two complexes as sensitizers in DSSCs.

The prepared N3–S dye showed comparable performance in DSSC to commercial N3. The lower conversion efficiency of N3–Se required further modifying the molecular structure of Se–N3 dye by incorporation of more electron donating groups in order to reduce its life time.

## Consent for publication

Authors approved consent on publication.

## Data Availability

All data generated or analysed during this study are included in this published article".
